# Insight of brain degenerative protein modifications in the pathology of neurodegeneration and dementia by proteomic profiling

**DOI:** 10.1186/s13041-016-0272-9

**Published:** 2016-11-03

**Authors:** Sunil S. Adav, Siu Kwan Sze

**Affiliations:** Division of Structural Biology and Biochemistry, School of Biological Sciences, Nanyang Technological University, 60 Nanyang Drive, Singapore, 637551 Singapore

**Keywords:** Dementia, Alzheimer disease, Neurodegenerative disease, Degenerative protein modifications (DPMs), Deamidation, Nitration, Oxidation, Biomarkers, β-amyloid

## Abstract

Dementia is a syndrome associated with a wide range of clinical features including progressive cognitive decline and patient inability to self-care. Due to rapidly increasing prevalence in aging society, dementia now confers a major economic, social, and healthcare burden throughout the world, and has therefore been identified as a public health priority by the World Health Organization. Previous studies have established dementia as a ‘proteinopathy’ caused by detrimental changes in brain protein structure and function that promote misfolding, aggregation, and deposition as insoluble amyloid plaques. Despite clear evidence that pathological cognitive decline is associated with degenerative protein modifications (DPMs) arising from spontaneous chemical modifications to amino acid side chains, the molecular mechanisms that promote brain DPMs formation remain poorly understood. However, the technical challenges associated with DPM analysis have recently become tractable due to powerful new proteomic techniques that facilitate detailed analysis of brain tissue damage over time. Recent studies have identified that neurodegenerative diseases are associated with the dysregulation of critical repair enzymes, as well as the misfolding, aggregation and accumulation of modified brain proteins. Future studies will further elucidate the mechanisms underlying dementia pathogenesis via the quantitative profiling of the human brain proteome and associated DPMs in distinct phases and subtypes of disease. This review summarizes recent developments in quantitative proteomic technologies, describes how these techniques have been applied to the study of dementia-linked changes in brain protein structure and function, and briefly outlines how these findings might be translated into novel clinical applications for dementia patients. In this review, only spontaneous protein modifications such as deamidation, oxidation, nitration glycation and carbamylation are reviewed and discussed.

## Introduction

Dementia is an increasingly common disorder of mental processes that confers memory loss, mood change, impaired reasoning, and eventual difficulty with day-to-day activities. According to World Health Organization’s (WHO) recent fact sheet (April 2016) [1], an estimated 47.5 million people worldwide already have dementia and there are approximately 7.7 million new cases being diagnosed each year. The total number of people with dementia is projected to exceed 75 million by 2030, and almost triple to 135.5 million cases by the year 2050. This exponential increase in the global prevalence of dementia, combined with its severe impact on patients’ families, caregivers and communities, have led the WHO to identify dementia as a major public health priority [2]. In 2010, the total global cost of dementia was estimated at $604 billion USD, corresponding to 1 % of worldwide gross domestic product. Indeed, due to the lack of effective prevention strategies and/or curative treatments, the social and economic costs of dementia disorders have surpassed those attributed to heart diseases and cancer [3]. Despite recognizing dementia as priority healthcare challenge throughout the world, funding allocation to dementia research still lags far behind expenditure on other major disorders. For example, in 2012 the UK allocated just 11 % of total research funding to dementia studies whereas cancer research received 64 % [4].

The most common forms of dementia are Alzheimer’s disease (AD) and Vascular dementia (VaD), which respectively account for 70 and 15 % of all dementia diagnoses [5], but the boundaries between dementia subtypes are not clear and mixed forms of these disorders are also thought to contribute to the total disease burden [6]. In recent decades, scientific study of dementia subtypes has failed to significantly improve our understanding of disease pathogenesis or generate effective new treatments or interventions for these disorders [7, 8]. At present, the mechanisms that initiate the disease process remain largely unknown and severely restricting attempts have been made to identify novel methods of disease prevention. Consequently, there remains an urgent need to better define the molecular basis of dementia pathogenesis and identify therapeutic targets that can prevent disease progression and/or alleviate symptoms in affected individuals.

**Fig. 1:**
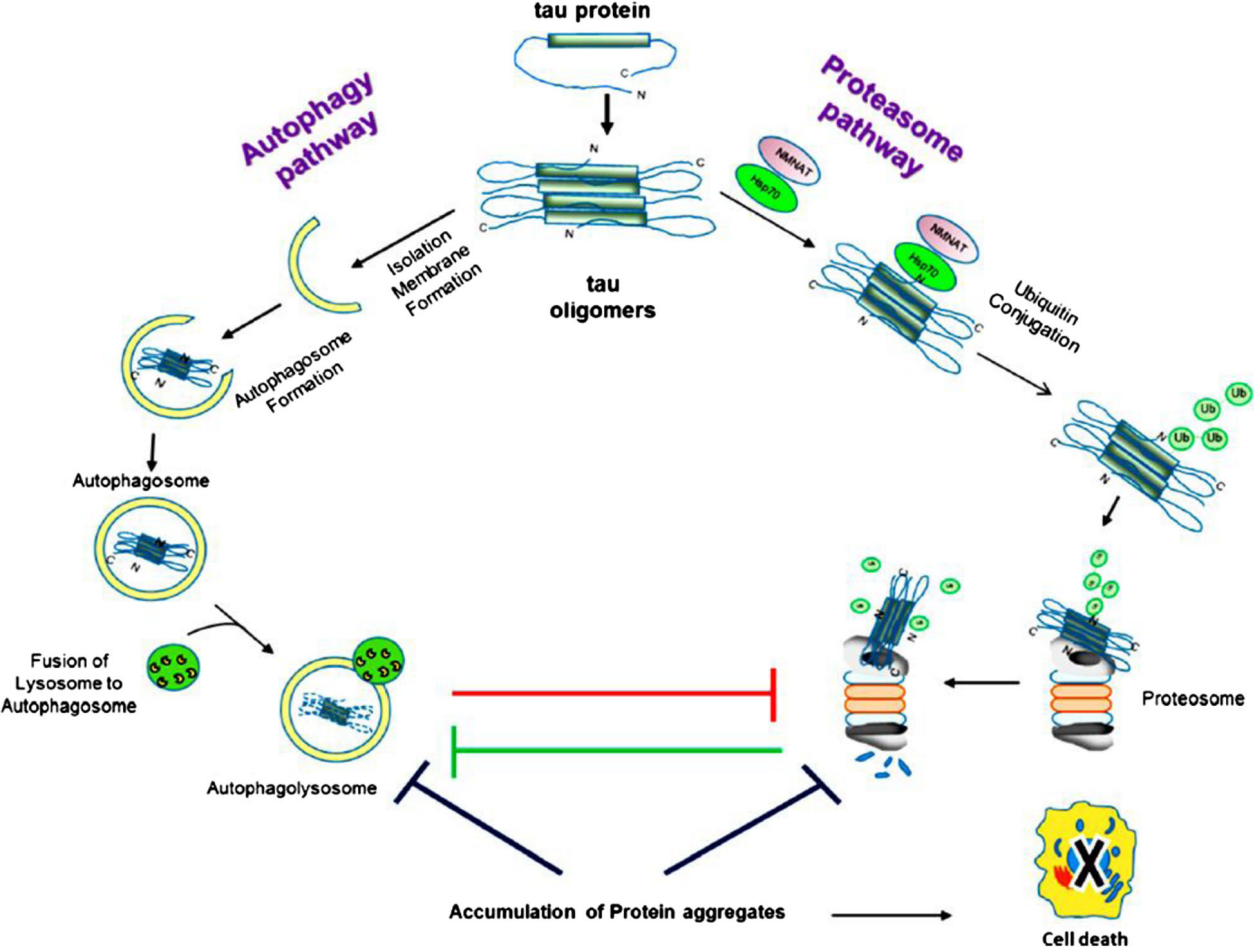
Tau oligomer turnover and principal clearance mechanisms. Impairment of one or both clearance pathways leads to protein accumulation and toxicity which impairs cellular function and eventually induces cell death. (Adapted from Cárdenas-Aguayo et al. [11])

Several models of dementia pathogenesis have been proposed since this syndrome was first reported over a century ago. Initial hypothesizes suggested that dementia was induced solely by ischemic cerebral vascular disease or stroke [9, 10]. However, the later discovery of aggregated β-amyloid (Aβ) and Tau proteins [11] in the brain tissues of dementia patients directed the majority of subsequent research effort towards the study of these two molecules alone. Accordingly, it was later proposed that dementia can be triggered by the toxicity of oligomerized proteins including Aβ and Tau which form senile plaques in the brain [11, 12]. According to this hypothesis, Tau proteins become toxic by forming paired helical filaments (PHFs) which are assembled into the neurofibrillary tangles (NFTs), characteristic of dementia pathology. However, Tau oligomers that form before PHFs and NFTs mediate dementia and neurodegeneration [11]. The known pathways and mechanisms involved in Tau oligomer clearance are depicted in Fig. 1. While substantial data have been presented in support of both hypotheses, the Aβ and Tau models each overlook several major aspects of dementia pathogenesis and have so far failed to yield a significant breakthrough in therapeutics. Indeed, while Aβ deposition is regarded as a hallmark feature and possible root cause of AD, the extent of Aβ deposition and senile plaques in the brain does not correlate with dementia severity, and healthy elderly individuals can exhibit abundant senile plaques even in the absence of AD [13–15]. The amyloid model of dementia pathogenesis therefore cannot fully explain disease initiation, progression, or clinical severity as observed in human patients.

Given that the burden of senile plaques in dementia patients does not correlate with cognitive dysfunction, the ‘proteinopathy’ underlying these disorders is likely to extend beyond the accumulation of Aβ and Tau protein. Accordingly, recent data have indicated that neurodegenerative diseases are further associated with DPMs [16–18] that confer loss of function and/or induce protein misfolding, aggregation ([19]), deposition, and degradation [20] in the brain. DPMs are spontaneous, non-enzymatic, posttranslational modifications caused by defective protein repair mechanisms and dysregulated protein turn-over. Increasing evidences from epidemiological, clinical, and experimental studies now suggests that cerebrovascular disease and hypoxia-ischemia injury in the brain are primary causes of the proteinopathy that leads to cognitive impairment and eventual dementia [10, 21–31]. While the pathology of these disorders is strongly associated with the deposition of complex protein aggregates in the brain, so far there have been few attempts to determine the roles of key proteins other than Aβ (or to identify the factors that first promote protein accumulation). Similarly, the contribution of protein DPMs to the initiation and progression of plaque formation remains largely unknown due to the technical challenges. More recently, state-of-the-art mass spectrometry-based proteomic techniques have allowed investigators to begin to address these issues by providing unprecedented power to detect critical changes in brain protein structure and function at the very earliest stages of dementia pathogenesis. Proteomic techniques will therefore be integral to uncovering the mechanisms that cause human neurodegeneration and dementia, as well as the identification of clinically useful prognostic biomarkers, and the design of novel interventions and therapies for affected patients. This review summarizes how the application of state-of-the-art proteomics technologies has provided novel insight into the molecular basis of dementia pathogenesis and the role of DPM-mediated protein alterations in tissue damage in the human brain.

### Proteomics of dementia disorders and Alzheimer’s disease

Dementia pathogenesis is typically divided into three main stages according to symptom and severity; early asymptomatic disease in which patients remain cognitively healthy, later mild cognitive impairment (MCI) due to accumulating tissue damage over time, and finally overt dementia with extensive pathology and disruption of normal brain functions [32]. It is important to note that dementia syndromes also include a number of distinct sub-pathologies that are each associated with damage to specific cell types in particular region of the brain (including AD, VaD, mixed dementia, Parkinson’s disease[PD], frontotemporal dementia [FTD], MCI, posterior cortical atrophy, traumatic brain injury, Down’s syndrome, Creutzfeldt-Jakob disease, and normal pressure hydrocephalus [33]). For example, one of the earliest symptoms of AD is a deficit in short-term recall due to tissue damage in the hippocampus, which is the brain center of learning and memory.

Dementia syndromes are highly heterogeneous and exhibit complex genetic associations. Current data suggest that early-onset dementia is caused by mutations in genes including amyloid precursor protein (APP), presenilin 1 (PSEN1), and presenilin 2 (PSEN2), leading to altered production of Aβ peptide, which is the principal component of senile plaques [34, 35]. Apolipoprotein E (Apo E) enhances deposition of Aβ in the brain, and the ε4 allele in particular is associated with increased pathology (homozygosity for this allele alone confers >8-fold increased risk of sporadic AD) [36]. Further, ApoE enhanced deposition of Aβ in the brain was validated using ApoE knockout mice [37] and crossed ApoE knockout mice with transgenic mice overexpressing a human mutant APP gene [38] using immunostaining, as well as thioflavine-S flurescence and Congo-red birefringency technique [39]. In addition to other variants, mutations in the MAPT gene that encodes Tau protein have been strongly linked with the pathogenesis of FTD, corticobasal degeneration (CBD), and other forms of dementia [40, 41]. While these genetic factors either alone or in combination clearly exert a major influence on dementia risk [42–44], their exact roles in the associated tissue pathology remain unclear, and their expression in the brain may not be sufficient to confer symptomatic disease.

Patient genotypes are essentially stable over time, and transcriptomics offers only limited information about protein expression levels, conformations, and modifications that occur in cells. In contrast, proteomics can generate detailed information on cellular protein expression dynamics and how these are influenced by complex environmental stimuli. This approach provides unprecedented scope to assess how brain cell function is altered during disease progression, and identify the key cellular pathways that promote pathology in affected tissues. Uncovering these key pathways and identifying their critical components will be essential for the development of effective new therapies for dementia patients. Proteomic approaches, therefore, have an important role to play in the study of dementia syndromes, since these techniques not only enable protein quantitation, but also the identification of key interacting partners and dynamic structural modifications, which exert major influences on protein distribution and function in human cells and tissues.

Recent advances in proteomic techniques have enabled comprehensive analysis of protein biology in a wide range of settings. A typical proteomic work-flow for identifying and profiling protein DPMs involves protein separation, trypsin digestion, LC-MS/MS analysis and database searches. In addition to label-free proteomic methods, isobaric tags for relative and absolute quantitation (iTRAQ) and tandem mass tag (TMT) protein labeling techniques are now widely-accepted approaches for quantitative profiling of proteins and their modified variants in both cell lines and clinical tissue samples [45–52]. The presence of aggregated protein plaques in the brain is a common clinical manifestation of dementia, but the specific molecular mechanisms underlying each disease subtype may be distinct. Distinguishing subtypes of dementia is difficult to achieve without access to well-characterized clinical samples of specific brain regions, efficient methods of isolating plaques and aggregated proteins from tissue samples, and reliable techniques for determining the composition of both soluble and aggregated proteins and their associated DPMs. To this end, our laboratory has recently developed a number of techniques for the enrichment and quantification of amyloidal proteins, as well as the robust determination of specific DPM events and their locations in diverse protein families [47, 48, 51–56].

### Technical advances in identification of amyloidal proteins and associated DPMs

**Fig. 2:**
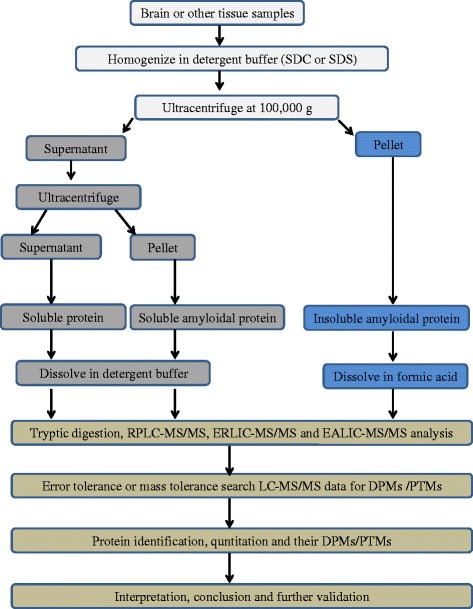
Flowchart summary of the isolation, identification and quantification of both soluble and insoluble amyloid proteins and their DPMs using a proteomic approach

Alterations in protein function and aggregation are key features of neurodegenerative diseases, but the factors that initiate and promote protein aggregation, accumulation, and deposition in insoluble plaques remain poorly defined. Due to their limited solubility and propensity for self-association, accurate identification and quantification of amyloidal plaque proteins in brain tissue extracts remains technically challenging. Previous attempts to isolate amyloidal proteins from human or rodent brain tissues have relied on the use of detergents or detergent-free buffers to perform sequential extraction and quantification by enzyme-linked immunosorbent assay (ELISA), immunoblotting, or immunocytochemistry [57, 58]. Du et al. [59] used quantitative *in vitro* kinetic aggregation assay that selectively, sensitively and quantitatively detect Aβ amyloid load in a variety of cell and tissue homogenates. Although different techniques like ELISA, immunoblotting, or immunocytochemistry were used to detect and quantify Aβ, these approaches were unable to fully elucidate either the composition or aggregation state of the constituent amyloids. The seminal studies utilized circular dichroism (CD) and NMR techniques to track the conversion of Aβ from soluble α-helical form to a fibrillar β-sheet protein [60]. As reviewed by Miller et al. [61], fourier transform infrared (FTIR) spectroscopy technique which is sensitive to the secondary structure of proteins can also be useful in investigating the process of protein misfolding and aggregate formation. In fact, techniques like x-ray crystallography and nuclear magnetic resonance (NMR) enable researchers to determine the three-dimensional structure of proteins; however, such techniques are not in the scope of this review. Recently developed, a novel proteomic approach based on ultracentrifugation-electrostatic repulsion hydrophilic interaction chromatography (UC-ERLIC)-coupled mass spectrometry made possible the detailed characterization of protein aggregates in human brain tissues affected by dementia [53]. Using a standard detergent buffer, this technique was able to successfully extract amyloids, soluble proteins, and insoluble aggregates from human brain tissues and identify dementia-associated changes in amyloid plaque composition, relative protein abundance, and extent of detrimental DPMs. Both the soluble proteins and amyloidal plaques were profiled using LC-MS/MS, which revealed that the insoluble aggregates were significantly enriched in proteins including S100A9, ferritin, hemoglobin subunits, collagen, and creatine kinase [53]. Intriguingly, plaque enrichment in S100A9 was attributable to the accumulation of the deamidated variant of this protein, suggesting a critical role of protein deamidation in the pathology of dementia. However, in this case report, authors used one patient without pathological confirmed degeneration and no analysis of tissue from control group remains as a major limitation. Further refinement of our previously reported protocol (Fig. 2) should enable future studies to improve the detection and identification of amyloidal proteins in human brain tissues [53].

Most types of DPM involve the addition of small chemical motifs to protein side [62] chain functional groups and confer minor shifts in overall mass [63]. These modifications cause alterations in peptide/protein charge and hydrophobicity, but due to their low abundance in the trypsin-digested protein sample, detection of these DPM-modified variants remains extremely challenging. However, by using an ion exchange column running in hydrophilic interaction liquid chromatography (HILIC) mode, the altered charge-state and hydrophilicities of the DPM-modified peptides make it possible to distinguish these from their unmodified counterparts via LC-MS/MS [56]. Moreover, the unmodified and modified peptides elute from the ion exchange column in a predictable order based on their charge densities in the LC-MS/MS mobile phase. Consequently, each of the peptide variants can be separated using electrostatic-interaction modified HILIC hydrophilic interaction liquid chromatography (emHILIC) methods together with weak anion exchange (WAX)/strong anion exchange (SAX) columns in ERLIC for online ERLIC-MS/MS analysis. Alternatively, peptide variants can be separated via the use of weak cation exchange (WCX) columns in electrostatic attraction hydrophilic interaction chromatographic mode (EALIC) for online EALIC-MS/MS analysis.

The extent of DPMs and PTMs of proteins in complex samples can be accurately quantified to infer their biological functions if the whole proteome of complex samples can be recorded in a single dataset without fractionation. A chromatographic strategy that uses a long (50 cm) anion-exchange capillary column operating in the electrostatic repulsion-hydrophilic interaction mode (LERLIC) and coupled directly to MS/MS has been developed for complex proteome analysis in a single injection [62]. The LERLIC-MS/MS method has been applied to resolve and quantify N- and Q-deamidation products, i.e. peptides containing iso-asp/asp or peptides containing γ-glu/α-glu isomers. Any deviation from the expected ratio (iso-asp/asp = 3 or γ-glu/α-glu =1.7) of spontaneous changes indicated enzymatic activities. The results from the study demonstrate that LERLIC-MS/MS can be used to perform an in-depth study of protein DPMs on a global proteome scale. This new strategy may be used to elucidate the biological implications of DPMs/PTMs in aging and disease conditions. The applications of different techniques along with their strengths and limitations have been tabulated in Table 1. Further proteomics techniques like top-down, bottom up with ECD/ETD and non-ECD methods for identifying DPMs such as deamidation were revived by Hao et al. [48].

**Table 1:** Summary of proteomics and chromatography techniques used in quantitative and qualitative proteomics

Technique	Details	Application	Strengths	Limitations
iTRAQ	Isobaric tags for relative and absolute quantitation	• Protein quantification through incorporation of stable isotopes • Isobaric tagging of peptides	• Multiplex several samples • Relative quantification • High-throughput	• Increases sample complexity • Require fractionation of peptides before MS
SILAC	Stable isotope labeling with amino acids in cell culture	• SILAC relies on metabolic incorporation of a given ‘light’ or ‘heavy’ form of the amino acid into the proteins, • Direct isotope labeling of cells Differential expression pattern	• Degree of labeling is significantly high • Quantitation is straightforward	• SILAC labeling of tissue samples is not possible
ICAT	Isotope-coded affinity tag	• Chemical isotope labeling for quantitative proteomics	Sensitive and reproducible Detect peptides with low expression levels	• Proteins without cysteine residues and acidic proteins are not detected
TMT	Tandem Mass Tag	• Protein quantification through incorporation of stable isotopes • Isobaric tagging of peptides	• Identification and quantitation of proteins in different samples • Relative quantification • Targeted quantitation strategies like SRM • High-throughput	• Increases sample complexity • Require fractionation of peptides before MS
HILIC	Hydrophilic Interaction Liquid Chromatography	• Analysis of charged substances • Separating polar proteins\peptides • Separation and quantitative analysis of modified and unmodified peptides	• The altered charge-state and hydrophilicities of the DPM-modified peptides make it possible to distinguish these from their unmodified counterparts via LC-MS/MS	• Longer column equilibration time, • Less reproducible retention times, • Higher cost of mobile phase
emHILIC	Electrostatic-interaction Modified HILIC hydrophilic interaction liquid chromatography	• Separation and quantitative analysis of modified peptides	• Efficient separation of modified peptides from unmodified via LC-MS/MS	• Some peptides may not dissolve well in high organic solvent (90%ACN)
ERLIC using WAX or SAX	Electrostatic-Repulsion Hydrophilic Interaction chromatography	• Separation of isoforms of peptides and proteins based on pI and hyrophobicities. • Study protein DPMs/PTMs to inferior their biological functions based on quantitation	• Quantitation of isoforms of peptides and proteins, e.g. the trios of deamidation products.	• Some peptides may not dissolve well in high organic solvent (90%ACN) • ERLIC chromatographic resolution is lower than C18 RP column.
LERIC-MS/MS	Long-length Column Electrostatic-Repulsion Hydrophilic Interaction chromatography coupled to tandem MS	• Study global protein DPMs/PTMs in whole complex proteomes like brain tissue lysate or cell lysate.	• Record the whole proteome in complex sample in a single LC-MS/MS data file for global DPMs/PTMs analysis.	• Some peptides may not dissolve well in high organic solvent (90%ACN). • ERLIC chromatographic resolution is lower than C18 RP column.

### Mass spectrometry based quantitative analysis of brain tissues in clinical settings

Use of stable isotope incorporation to facilitate relative quantification of proteins has become a vital technology in modern proteomic research. In a previous study, the temporal cortex of patients with pathologically-confirmed VaD was compared with matched control brain tissues using a 2D liquid chromatography-coupled tandem mass spectrometry-based iTRAQ technique (2D-LC-MS/MS-iTRAQ) [46]. In this report, proteomic profiling of the specific brain region known as Brodmann area (BA) 21 revealed VaD-associated up-regulation of 144 proteins including superoxide dismutase, neural cell adhesion molecule, and ATP synthase subunit α, suggesting a state of hypometabolism, vascular insufficiency, and tissue inflammation. These proteomics results were further validated using western blot analysis by selecting proteins involved in different pathways such as energy metabolism (e.g. ATP5A, UQCRC2), oxidative stress (e.g. SOD1, ferritin), inflammation (e.g. NCAM, ICAM5), synaptic transmission (e.g. SYNPO, syntaxin) and apoptosis (e.g. HSPA4, PEA15, VDAC1), oxidative phosphorylation (i.e. SDHB, MT-CO2 and NDUFB8). Furthermore, iTRAQ quantitative proteomic analysis of brain samples from VaD subjects also indicated significant down-regulation of ion channel proteins including V-type proton ATPase subunits D and F, Obg-like ATPase 1, and ATP5F1 (ATP synthase, H^+^ transporting, mitochondrial F0 complex, subunit b) [45]. Using proteomics and structural modeling of the multi-functional ion channel protein Na^+^-K^+^-ATPase, Sze and coworkers proposed that impaired regulation and function of Na^+^-K^+^-ATPase contributes to the pathophysiology of VaD [45]. These data are consistent with the known role of Na^+^-K^+^-ATPase in maintaining differential membrane potential in neurons for effective signal transduction, as well as reports that Na^+^-K^+^-ATPase expression and/or function are dysregulated in both disease models and brain tissues from dementia patients [64].

**Fig. 3:**
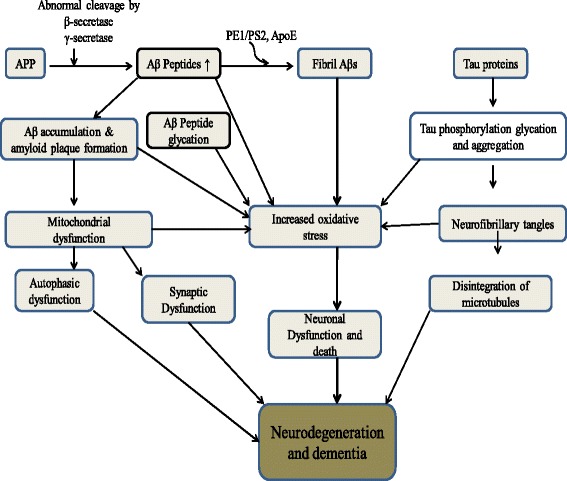
Proposed roles of amyloid precursor protein, specific gene mutations, and various DPMs in human neurodegeneration and dementia

Synaptic failure is the most common feature observed in both VaD and AD, and loss of synapses and/or synaptic contacts is a critical determinant of cognitive impairment in VaD and other neurodegenerative diseases [51, 65]. Similarly, a decline in synapse number in the hippocampal dentate gyrus has been reported to correlate with impaired performance during cognitive testing in AD [66]. These data suggest that hippocampal degeneration is central to pathological memory loss in AD. Another archetypal feature of AD is mitochondrial dysfunction, although the underlying basis of this defect remains unclear. One of the many current hypotheses (summarized in Fig. 3) suggests that mitochondrial dysfunction in neurodegenerative disorders results in the generation of reactive oxygen species and oxidative stress, thereby inducing DPMs that impair protein function and promote aggregation in affected tissues. For example, Caspersen et al. used transgenic mice expressing human mutant amyloid precursor protein (mAPP) to demonstrate that accumulation of Aβ in brain mitochondria impairs neuronal function and promotes cellular dysfunction [67]. Consistent with these data are reports that the early stages of AD are characterized by a reduction in neuronal mitochondria and decreased brain metabolism of glucose [68, 69]. As reviewed by Butterfield et al., analysis of autopsied AD brain tissues also revealed decreased pyruvate dehydrogenase activity in the parietal, temporal, and frontal cortex, as well as reduced activity of cytochrome c oxidase and mitochondrial complex IV [70].

**Fig. 4:**
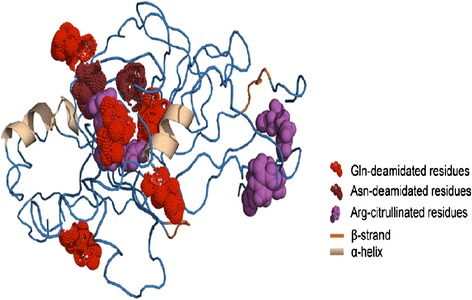
Citrullination of arginine and deamidation of glutamine in myelin basic protein from female patients with AD-CVD (adapted from Gallart-Palau et al. [55])

Gender may be an important modifying factor in the development and progression of dementia, but gender differences in neuropsychological functions are seldom evaluated. Pusswald et al*.* assessed gender-specific differences in cognitive dysfunction between male and female patients with mild to moderate AD, and they observed that disruption of verbal learning in early-stage disease was more pronounced in women than in men [71]. These data are in-line with an earlier meta-analysis of neurocognitive data pooled from 15 independent studies of AD patients (*n* = 828 men; 1238 women), which revealed that male patients significantly outperform female patients when tested for verbal and visuospatial ability as well as recall of episodic and semantic memory [72]. Other researchers have also reported that females are more vulnerable to dementia than males [73], and epidemiological data indicate that AD is more prevalent in women than in men [74], but the mechanistic basis of this gender bias remains largely unknown. However, recent quantitative analyses of brain protein expression in dementia patients have begun to shed light on this phenomenon. In proteomic study of frontal cortex tissues from AD patients, Muller et al. observed that disease-associated up-regulation of heat shock 70 kDa protein 1B and glyceraldehyde 3-phosphate dehydrogenase (GAPDH) is gender dependent [75]. Similarly, data from our own laboratory have revealed significant modulation of several redox proteins in the temporal lobe of Alzheimer’s disease with cerebrovascular diseases (AD-CVD) patients as well as sex-specific alterations in the white matter and mitochondrial proteome of female patients [55]. Proteomics findings of up-regulation of myelin proteolipid protein (PLP) and their enrichment in the temporal lobe of female AD-CVD was further validated by western blot techniques [55]. Proteomic analysis of AD-CVD brain tissues suggested that myelin basic protein (MBP) exhibited hyper-citrullination of arginine and deamidation of glutamine only in female patients (Fig. 4). These data are consistent with reports that down-regulation of cathepsin D and other enzymes that degrade damaged brain proteins can enhance citrullination of MBP, leading to axonal dysregulation and progressive loss of neuron function [76].

### Mass spectrometry based proteomic insight into hypoxia/ischemia-induced neuropathies

Decreased cerebral blood flow has been correlated with the symptoms of dementia in both MCI and early-phase AD [77]. In the affected tissues, decreased blood flow triggers cellular hypoxia, which has been implicated in the pathogenesis of AD [78]. Unbiased proteomic analysis has provided novel insight into the molecular pathology of hypoxic-ischemic brain injury, and confirmed that low-oxygen conditions can induce mitochondrial dysfunction and cellular stress as well as pathological epigenetic changes and dysregulated proteostasis [45, 47, 51, 79, 80]. Oxygen restriction has also been observed to induce specific pathological changes in neuronal cell lines [79], rodent models of cardiovascular disease and cerebral artery occlusion [80, 81], and in brain samples from dementia patients [45, 47, 51], suggesting a likely central role for hypoxia-triggered pathways in driving progressive tissue damage and corresponding cognitive decline. For example, a previous proteomic study of ischemic neuronal injury identified significant dysregulation of proteins including PARK7 and VAP-A that have already been implicated in the pathology of chronic neurological disorders such as AD and PD [79]. Similarly, when an iTRAQ proteomic approach was used to assess the neuronal cell response to hypoxia and glucose depletion stress in a hypoxic-ischemic penumbra model, the authors detected substantial dysregulation of multiple housekeeping proteins, as well as mediators of anti-oxidative defence, chaperone functions, and protein metabolic pathways [79]. Further, these authors adopted functional and cytometric assays, as well as western blotting technique to validate their iTRAQ-quntitative proteomics findings. Proteomic methods have also been used to uncover the molecular basis of progression from hypoxia-ischemia brain injury to overt clinical dementia, which is associated with dysregulation of energy metabolism, mitochondrial dysfunction, neuro-inflammation, and synaptic failure [45–47, 82]. Consistent with these data, other reports have observed decreased activity of α-ketoglutarate dehydrogenase and impaired operation of the Krebs cycle in AD brain [83, 84]. The role of hypoxia and impact of neurotransmitter γ-aminobutyric acid (GABA) shunting in the pathogenesis of AD has recently been reviewed by Salminen et al. [83]. Taken together, these data strongly support the concept that neurodegeneration is caused by a successive cycles of hypoxic-ischemic brain injury which induce detrimental DPMs that promote protein misfolding and aggregation, leading to cognitive decline and eventual dementia. While the molecular events that drive early subclinical proteinopathy in the brain remain poorly understood, a likely key mediator is the hypoxia-inducible transcription factor HIF, which potently modulates cellular gene expression upon stabilization under low-oxygen conditions. Following recent technological advances, it will now be possible to determine the exact role played by HIF and other potential mediators of human proteinopathies using unbiased, global, discovery-driven proteomic approaches. Our group and other researchers therefore optimized the use of proteomic techniques for the systematic analysis of hypoxia effects on neuronal cell lines, animal models of ischemic brain injury, human blood plasma samples, and post-mortem brain tissue samples from patients affected by dementia or stroke [79–82, 85–89]. Using this approach, researchers have achieved good progress in understanding how protein DPMs and aggregation induced by hypoxic-ischemic brain injury can promote neurodegeneration in dementia [90–92].

### Degenerative protein modifications and their impact in dementia

DPMs critically regulate a wide range of homeostatic and pathological processes by modulating protein activity, charge, hydrophobicity, stability, specificity, transport, and longevity in human cells [93, 94]. By combining chromatography with mass spectrometry, it is now possible to conduct robust identification and characterization of novel DPMs using proteomics platforms, which offer high sensitivity of detection, accurate assignment of structural modifications, and robust quantification of changes in DPM-bearing amino acids at specific locations [47, 48, 95]. When applied to the analysis of DPMs in complex biological samples, proteomic approaches provide unparallel power to assess the molecular basis of ‘proteinopathies’ such as dementia. Consequently, we now recognize that DPMs can promote pathological progression in human dementia by radically altering protein structure and function in the brain [45, 55]. There are several major types of DPM known to occur in human cells; deamidation, phosphorylation, nitrosylation, glycosylation, racemization, glycation, and hydroxylation, although these are not the only known modes of modification. While some DPMs are catalyzed by enzymes, this review focuses on the spontaneous (non-enzymatic) modifications such as deamidation, oxidation, nitration, carbamylation and glycation; which are recently identified as being key mediators of brain proteinopathy in dementia.

### Protein deamidation in dementia disorders

Cellular degradation of modified proteins is required to avoid the accumulation of altered/non-functional molecules and protect against neurodegeneration and dementia. Conversely, increasingly prevalence of specific DPMs in the brain may serve as useful biomarkers of disease, such as elevated tissue levels of hyper-phosphorylated Tau, which has previously been used in the diagnosis of dementia [96]. While multiple types of DPMs are now known to promote protein misfolding, aggregation and accumulation in the brain, the initiating factors and mechanisms that mediate these DPMs remain poorly understood, primarily due to the technical challenges associated with their study. To define the role of specific DPMs, it is first necessary to perform accurate identification of protein modification sites, while avoiding introduction of artificial modifications during sample preparation. Processing proteomic samples at a mild alkaline pH together with prolonged trypsin digestion at 37 °C are major causes of non-enzymatic Asn-deamidation, but use of an alternative protocol in which trypsin digestion is conducted in 50 mM ammonium acetate (pH 6) can mitigate artifactual deamidation, improve sensitivity, and increase confidence of identifying low abundance DPMs [48, 52]. Recovery of low-abundance peptides can also be further improved via the use of sodium deoxycholate (SDC) with an ammonium acetate-based buffer (pH 6.5) which has been shown to increase protein solubility and enhance trypsin activity during the processing of complex biological samples [97]. By avoiding the use of urea and employing mildly acidic conditions, this approach was able to limit artificial asparagine deamidation and prevented artifactual carbamylation [97].

Under physiological conditions, deamidation of the protein residues asparagine (Asn) and glutamine (Gln) can occur spontaneously and progressively alter protein structure, function, and stability over time [98]. Asn deamidation occurs through the formation of a succinimide ring intermediate, which is quickly hydrolyzed to D,L-Asp and D,L-isoAsp (with isoAsp predominating). Due to the less favorable thermodynamics of forming a six-member glutarimide ring, deamidation of Asn occurs relatively frequently whereas Gln deamidation occurs more slowly. Deamidation increases peptide mass by just 0.984 Da, and the hydrophobic properties of the resultant Asp- and isoAsp-containing variants are extremely similar, hence it is challenging to resolve these using conventional MS-based techniques. However, by developing an improved ERLIC-LC-MS/MS methodology, it has recently become possible to distinguish isoAsp-containing peptides from their n-Asp-containing counterparts prior to identification. While protein deamidation can serves as a versatile molecular clock that regulates many normal cellular processes, excess DPM accumulation in long-lived proteins can eventually lead to age-related decline in biological function [48]. For example, deamidation has previously been linked with progressive alterations in the structure of human cortical neurons, as well as the accumulation of α-synuclein protein in patients with PD, AD, multiple system atrophy (MSA), or dementia with Lewy bodies (DLB) [99]. Increased levels of isoAsp have also been detected in AD brain compared with healthy subjects, and are enriched in Aβ peptides isolated from amyloid plaques [100]. Excessive deposition of isoAsp residues has also been identified in synapsin 1 and tubulin proteins in VaD patients [51], strongly suggesting that deamidation impairs synapse protein function early in dementia pathogenesis.

**Fig. 5:**
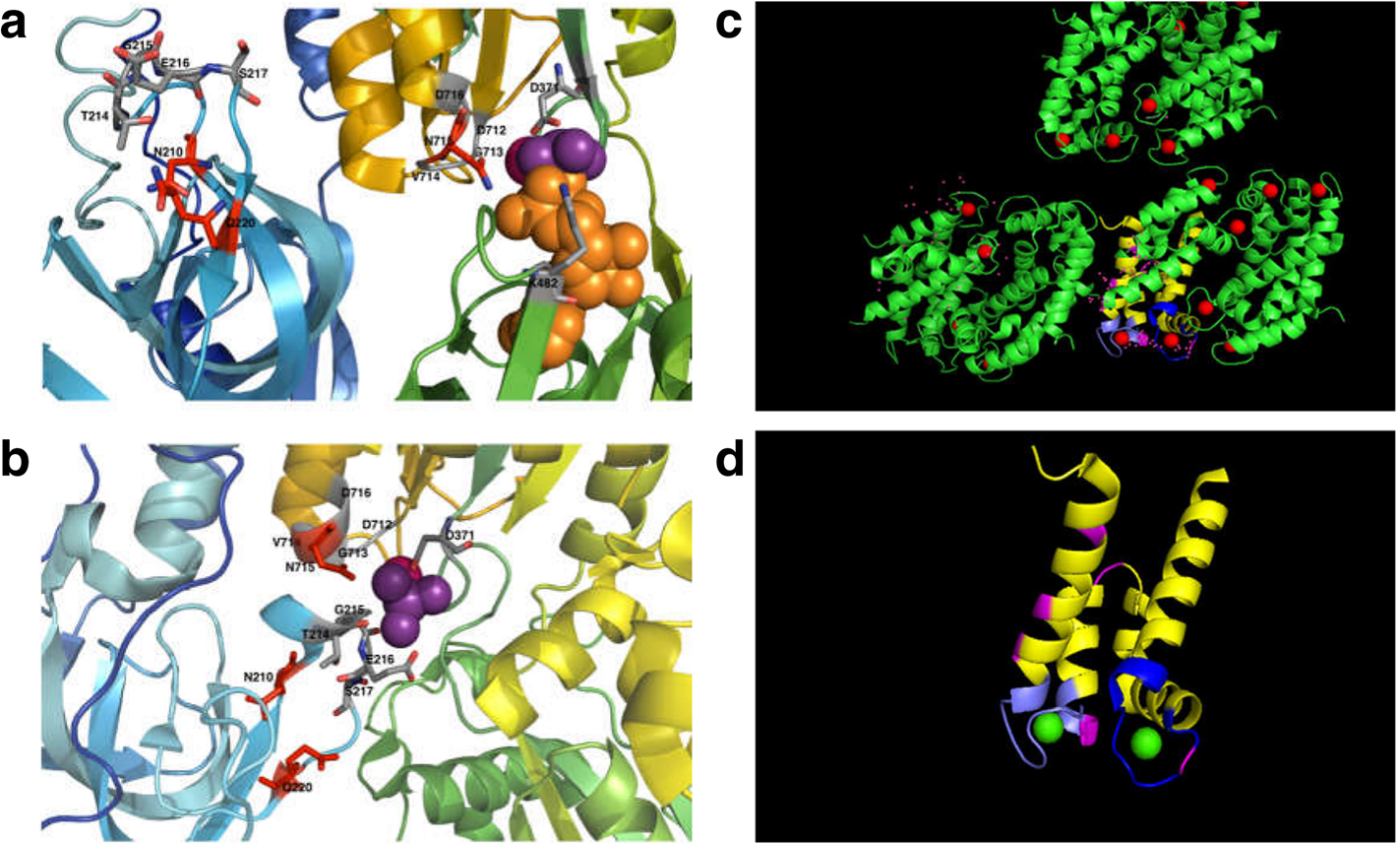
Structural models of the Na^+^/K^+^-ATPase catalytic site in (**a**) E_1_P and (**b**) E_2_P conformations (PDB ID 4HQJ and 2ZXE respectively). In both **a** and **b**, color-codingshows in blue: the amino-terminal; red: carboxyl-terminal, blue and cyan: A domain; yellow: P domain; green: N domain. Deamidated residues N210, D220 and N715 are highlighted in red. Magnesium ions are represented in magenta. (Adapted from Adav *et al.* [45]). **c** Structural model of deamidation sitesin protein S100A9 (RCSB Protein Data Bank accession code: 1XK4). The EF hand calcium binding motifs are shown in yellow and deamidation sites are highlighted in magenta and blue. **d** EF hands and deamidation sites (adapted from Adav *et al.* [53])

The ion channel protein Na^+^–K^+^ ATPase exhibits multiple functions including the maintenance of differential membrane potential in neurons, which is an essential feature of signal transduction processes. Dysregulation of Na^+^–K^+^ ATPase expression or function have been reported in both animal models and human brain tissues affected by AD, PD, or HD [46, 79, 81, 82]. In a previous study of human brain tissues from patients with VaD, Adav et al. observed deamidation of Na^+^–K^+^ ATPase subunits in evolutionary conserved regions of the protein (Fig. 5a, b) [45]. Using a structural modelling approach, they then located the specific modification sites and proposed that disruption of Mg^2+^ and Cu^2+^ binding impaired electrostatic interactions and inhibited the function of ion channel proteins in VaD. Modification of Na^+^–K^+^ ATPase residues 210 and 220 has been proposed to cause defects in protein phosphorylation and dephosphorylation mechanisms, potentially leading to altered ATP hydrolysis in the brain [45, 101, 102], and ATP synthase α-chain was previously reported to accumulate in the cytosol in early stages of neurofibrilliary degeneration in AD [103]. Deamidation-induced changes in Na^+^–K^+^ ATPase subunits may therefore lead to defects in membrane excitability and neuronal function. Moreover, the protein ‘L-isoaspartate (D-aspartate) O-methyltransferase’(PIMT) functions as a repair enzyme that can recognize abnormal isoAsp residues and restore them to the unmodified L-Asp form, thus allowing deamidation to be reversed. However, a previous proteomic analysis of VaD brain tissues revealed that PIMT is also deamidated in this condition, likely resulting in reduced capacity to mediate repair of isoAsp residues [45]. In mammalian cells and mouse models that lack PIMT, isoAsp accumulation causes hyperactivation of key cell signaling pathways, decreases animal growth, and can even induce fatal seizures [104].

During a previous study of dementia-linked amyloidosis, analysis of the pelleted insoluble aggregate revealed extensive deamidation of brain proteins including S100A9, ferritin, hemoglobin, creatine kinase (U-type), S100-B, collagen α-2(IV) chain, collagen α-2(I) chain, laminin subunit β-2, dystonin (isoform 3), and serine/threonine-protein kinase (isoform 2) [53]. These authors also observed disease-associated deamidation of the proteins coronin-1A and syntaxin-binding protein 2, which have previously been implicated in neurodegeneration in the hippocampus. Since deamidation introduces a negative charge that promotes protein aggregation, the multiple deamidated residues of S100A9 (Fig. 5c and d) suggest a propensity to modify charge state and form pathological aggregates in the brain. Similarly, loss of synapses is a major contributor to the cognitive impairments that is manifested in VaD, and temporal cortices from affected patients exhibit upregulation of the synaptic protein SNAP25 (synaptosomal-associated protein 25) [47], as well as significant accumulation of deamidated asparagine and glutamine residues in nerve terminal protein synapsin 1 compared with age-matched controls [51]. When assessed using structural models, the location of the modification site predicted that deamidation would induce detrimental changes in the conformation of synapsin 1. Accurate identification of DPMs and the affected modification sites will therefore be critical to understanding their influence on protein biology in human dementia. Efforts have therefore been made to develop a comprehensive approach to the accurate identification of protein DPMs in the brain, including method optimization for biomedical and clinical applications [45, 47, 48, 51–53, 56].

### Protein oxidation in dementia disorders

Protein oxidation is thought to represent the primary mode of molecular aging since this modification not only directly damages the target protein, but also generates additional reactive metabolites that promote further oxidation of cellular proteins, lipids, and nucleic acids. Under physiological conditions, healthy cells and tissues maintain a balance between pro- and anti-oxidative mediators, but in disease settings this balance gets disrupted and permit increased production of reactive nitrogen species (RNS), formation of advanced glycation end product (AGEs), and generation of reactive oxygen species (ROS) including superoxide anions (O_2_^•-^) and hydroxyl radicals (OH^•^). Various physiological processes can also produce singlet oxygen and hydrogen peroxide (H_2_O_2_) which destabilize lysine and histidine residues, promote cysteine conversion into disulfides, and enhance the formation of methionine sulfoxides. The resultant oxidative stress can impair vital cellular processes, signalling events, and metabolic pathways, thereby contributing to the pathogenesis of multiple neurodegenerative disorders [105–107]. Oxidated proteins may also exhibit altered sensitivity to proteolytic degradation and increased tendency to form insoluble aggregates in the brain. Accordingly, oxidative protein damage has already been implicated in the pathogenesis of AD, PD, Huntington’s disease (HD), DLB, and ALS [108, 109].

Oxidative damage has previously been linked with protein loss-of-function in the hippocampus, which is strongly associated with memory impairment and cognitive dysfunction in dementia [108, 110, 111]. While multiple proteins are known to be subject to oxidative modification in AD brain tissues (including enolase, TPI, PGM1, CK, LDH, GAPDH, aconitase, aldolase, VDAC, and ATP synthase [108, 112–114]) a previous region-specific analysis by Sultana et al. determined that the hippocampus was specifically enriched in oxidated variants of ubiquitin carboxy-terminal hydrolase L-1 (UCH L-1), peptidyl prolyl cis–trans isomerase, phosphoglycerate mutase 1, dihydropyrimidinase-related protein2, carbonic anhydrase II, triose phosphate isomerase, α-enolase, and γ-SNAP [108]. These data are consistent with reports that brain protein oxidation preferentially occurs in Aβ-rich regions including inferior parietal lobe (IPL), cortex, and hippocampus, but not in the cerebellum which typically contains only trace levels of Aβ [110]. In-line with these findings, other authors have used proteomics-coupled 2D fingerprinting with immunological detection of carbonyls to confirm that IPL tissues from AD patients are enriched in oxidated proteins that play crucial roles in energy production, axonal growth, pH regulation, vesicular transport and protein degradation [108, 109, 113, 115]. The oxidated proteins included CKBB, which plays a major role in the production of high-energy phosphate for ATP synthesis, the ubiquitin-proteasome component UCH L-1, and the glutamate-glutamine cycle regulator GS which maintains the balance of neurotransmitters in the brain. Accordingly, oxidative modification of these proteins in AD patients has been reported to disrupt energy generation in brain tissues, impair synaptic function, and induce excitotoxicity-mediated neuronal cell death and consequent memory loss [113, 116–118].

Given that glucose is the primary energy source used in the brain [119], disruption of glycolytic metabolism, in particular, can exert profound effects on cognitive function. Several components of the glycolysis pathway may exhibit oxidation-related functional impairment in dementia, including enolase, TPI, PGM1, LDH, GAPDH [110, 112–114]. The corresponding deficiency in energy production not only disrupts normal synaptic functions, but also impairs ion motive ATPases, induces cholinergic defects, disturbs cholesterol homeostasis, and modulates protein synthesis and signal transduction pathways. Collectively, these effects can severely impact neuronal cell survival and memory functions, leading to progressive cognitive decline [109]. Inefficient neurotransmission has also been linked with oxidation of key proteins involved in production of the critical neurotransmitter acetylcholine (e.g. RAF kinase inhibitor, phosphatidylethanolamine binding protein, hippocampal cholinergic neurostimulating protein) [120]. Other detrimental effects of oxidation have been identified among brain cell structural proteins including β-actin, dihydroxypyrimidine related protein-2, and α-tubulin, with modification of these molecules being linked to a decline in interneuronal connectivity, impaired axonal transportation, and loss of structural integrity leading to neuronal cell death and AD-like pathology [121–123].

### Protein nitration in dementia and AD pathogenesis

While several mechanisms have already been proposed to trigger AD pathogenesis in brain tissues, it remains unclear what factors first cause loss of synaptic connections and memory loss in human patients. Brain tissue accumulation of amyloidal plaques, *Tau*, presenilin, apolipoprotein, and plasmin appear to be key contributors to disease development, but accumulating evidence increasingly points towards oxidative stress as a key initiator of AD pathogenesis [70, 105, 106, 117, 121, 124]. It is important to note that the effects of oxidative stress are not limited to proteins alone and can further modify cellular function via oxidation of other biomolecules, formation of advanced glycation end products, and generation of ROS/RNS. For example, while nitric oxide (•NO) can serve as a neurotransmitter and signaling molecule in healthy tissues, excess generation of •NO instead favors the formation of reactive peroxynitrite and nitrogen dioxide species that can mediate nitration of brain proteins [125]. Protein nitration involves the formation of NO_2_-Tyr motif that confers a characteristic mass increase of 45 Da, and has previously been reported to enhance Aβ aggregation in a rodent model of AD [125]. By mediating tyrosine nitration at the ortho-position, peroxynitrite modification of proteins can block later phosphorylation events, thereby inducing protein dysfunction and eventual cell death [126]. However, these events may not be wholly pathological, since other investigators have proposed that dynamic interplay between nitration and phosphorylation may be required for some normal biological functions [127], and that tyrosine nitration can contribute to neurite elongation and differentiation of neuronal cell types [128]. While peroxynitrite reacts directly with cysteine, methionine and tryptophan, modification of tyrosine, phenylalanine and histidine residues is instead driven by intermediary secondary species [129]. For instance, reactive nitrogen species formed by the combination of superoxide (O^2^•^-^) and nitric oxide (NO•) radicals can promote the formation of nitrothiols which inactivate mitochondrial enzymes and further modify membrane and cytosolic proteins to disrupt essential cellular functions [126, 130, 131].

Under normal physiological conditions, Tau is a natively unfolded protein with high solubility, but in AD brain tissues this protein undergoes modifications that induce changes in conformation and reduce solubility. Tau contains five tyrosine residues (located at 18, 29, 197, 310, and 394), that can undergo nitration to initiate a range of ‘tauopathies’ [132]. In AD patients, Tau nitration occurs selectively at Y18 and Y29, and to lesser extent at Y197 and Y394 [133]. Tau nitration at Y197 and Y18 has been reported to enhance disease progression in a range of neurodegenerative disorders [134], whereas nitration at Y29 appears to be a specific characteristic of AD [133]. Similarly, the abundant neuronal protein α-synuclein has been observed to form intracellular aggregates in patients with AD, PD or various ‘synucleinopathies’, perhaps as a consequence of protein nitration at tyrosine residues Y39, Y125, Y133 and Y136. Using a highly novel approach, Burai et al. assessed how site-specific incorporation of 3-nitrotyrosine into different regions of α-synuclein exerted diverse effects on protein structure, function, oligomerization, and aggregation [135]. The effects of protein nitration may therefore be as diverse as the range of targets that can undergo this modification. Indeed, the repertoire of proteins reported to be nitrated in AD has expanded to include many critical mediators of essential cellular functions, such as aldolases A and C, peroxiredoxin 2, neuropolypeptide H3, glutamate dehydrogenase, phosphoglycerate mutase1, TPI, and H^+^-transporting ATPase [114, 136, 137]. The AD hippocampus in particular also displays extensive nitration of α-enolase, GAPDH, carbonic anhydrase II, ATP synthase α-chain, and VDAC-1 [108]. As outlined in the previous section on protein oxidation, any modification that impairs glucose metabolism can potentially exert major effects on normal brain functions, and accordingly, nitration of ATP synthase α-chain and VDAC-1 are strongly associated with mitochondrial dysfunction and neuronal cell death in the hippocampus in AD.

While nitrated proteins are typically prone to proteosomal degradation, in AD patients this pathway may be defective due to oxidation of key components such as UCH L-1 in the hippocampus and IPL regions [114, 138]. Consequently, nitrated proteins may be able to escape degradation and instead accumulate to pathological levels in AD brain tissues. Indeed, elevated levels of 3-nitrotyrosine (NT) have been detected in neurons derived from AD brain tissues, together with increased concentrations of dityrosine and 3-nitrotyrosine in the hippocampus, IPL, and neocortical regions [139, 140]. It is important to note that when Su et al. examined NT levels in the visual cortex of AD patients, the authors observed that brain neurons can exhibit DNA damage in the absence of tangle formation, strongly suggesting that oxidative damage is an early event in the pathogenesis of AD [140].

### Protein glycation in dementia disorders

Glycation is a non-enzymatic process initiated by the reaction between a reducing sugar and the free amino group of a target protein in Maillard reactions or glycosylation events. The product of this reaction is known as an Amadori-modified protein, which can subsequently generate AGEs upon further modification by oxidation and fragmentation. Maillard reactions primarily occur at the ε-amino groups of lysine or at their free amino groups, whereas glycation can also take place at the side chains of arginine, histidine, tryptophan and cysteine residues [141]. Glycosylation is instead an enzyme-directed process that attaches glycans to proteins, lipids, and other organic molecules in a site-specific manner. Since this review article is focused on spontaneous protein modifications, the subsequent section will discuss only the non-enzymatic pathway of advanced glycation.

AGEs alter protein charge and solubility, as well as inducing conformational changes that promote the formation of insoluble protein deposits, increase oxidative stress, and elicit inflammatory responses in the brain [142, 143]. The proteins most vulnerable to AGE modification are those with slow turnover rates, such as fibronectin, collagen types III, IV, VI, laminin, and crystalline [144, 145]. Other long-lived proteins such as β-amyloidal plaque components are also susceptible to AGE modification, and increased levels of AGEs have been identified in tissues from patients with AD [146, 147]. The effects of protein glycation vary between target molecules, but a role in promoting aggregation and dysfunction of Tau has been well documented [148]. Tau is involved in stabilizing the neuronal cytoskeleton by interacting with microtubules, but glycation of this protein within the tubulin binding motif has been proposed to disrupt this role and impair neuronal functions [149, 150]. Tau exhibits six different isomers in adult humans, and protein function is critically regulated by the expression ratio and phosphorylation state of these variants. Accordingly, an altered ratio of Tau isomers has been linked with neurofibrillary pathology and neurodegeneration [151], and glycation of these variants in AD promotes phosphorylation events that disturb the normal isoform balance in the brain [148]. Tau exhibits increased propensity to form aggregates upon glycation or phosphorylation, which in turn promotes pathological bundling of cytoskeletal polymers [152, 153]. As well as being resistant to degradation, glycated proteins are also highly susceptible to oxidation and support further generation of damaging free radicals, leading to brain tissue accumulation of these molecules and eventual neuronal cell death [150, 154–156].

AGE-modification accelerates the aggregation of soluble Aβ proteins *in vitro*, and AGE adducts are enriched at least 3-fold in plaque samples from AD brains compared with age-matched controls [157, 158]. Brain enrichment of AGEs may impair neural cell function by promoting covalent cross-linking of cellular proteins, thereby impeding their normal activities, or alternatively by direct signalling through the receptor for AGE (RAGE) [159]. Aβ has previously been identified as a ligand for RAGE, and upregulation of RAGE has been reported to mediate Aβ-induced oxidative stress, activate NF-kB, promote neuronal expression of macrophage colony-stimulating factor, and induce neuronal dysfunction [160, 161]. A previous study by Fang et al. also observed that RAGE signalling in microglia elicits an inflammatory response that impairs neuronal function and directly influences amyloid accumulation [162]. As well as inducing expression of pro-inflammatory cytokines, AGEs have also proposed to contribute to the development of VaD [163], thus suggesting a critical role for this modification in pathological cognitive decline. However, AGE-induced cross-linking and denaturation of cellular proteins may also represent part of the normal aging process [164], hence further study will be required to distinguish pathological events from natural processes.

### Protein carbamylation in dementia disorder

Carbamylation, a nonenzymatic DPM mediated by binding of cyanate derived from urea dissociation or myeloperoxidase-mediated catabolism of thiocyanate to free amino groups of proteins, impair function of protein and thought to promote vascular dysfunction during end-stage renal disease [165, 166]. Carbamylation may result in changes in the properties of the proteins, ranging from complete loss of biological activity to minor conformational effects and even increase in activity. In 1970s, Crist et al. [167], noted a dose-based decline in learning ability when rats were injected with 50, 100 and 150 pmol cyanate per day, while in vivo and in vitro evidences of brain protein carbamylation by cyanate was documented by Fando and Grisolia [168]. Recently, it’s been documented that carbamylation promotes molecular aging through alteration of protein functions, especially long-lived extracellular matrix proteins [169]. But, recent literature on protein carbamylation in dementia and AD disorder is very limited, may be due to technical challenges like firstly, urea used as a denaturant for sample preparation induces artifactual carbamylation, and secondly, a mass shift of +43 Da (carbamylation) is difficult to distinguish from +42 Da (trimethylation or acetylation), and apparently real identifications of carbamylation could potentially be artifactual [170]. However, upon kidney function decline, the urea accumulates elevating the burden of carbamylation. After proposing the carbamylation of erythropoietin (CEPO) for removing its erythropoietic effects, several researchers [171–174] carried out preliminary experiments signifying the neuroprotective effects of CEPO in a wide range of animal models of neurotoxicity including ischemic stroke, sciatic nerve compression, spinal cord depression, and peripheral diabetic neuropathy and proposed CEPO as a potentially important pharmacological agent for the treatment of neuropsychological disorders, neuronal function and chronic neuronal disorders including AD, PD, HD, amyotropic lateral sclerosis, multiple sclerosis, Creutzfeldt–Jakob disease, Charcot–Marie Tooth Disease, ataxias, seizure disorders, stroke, brain or spinal cord trauma [171, 173, 175]. The major structural proteins of the eye lens called α-crystallin functions like a chaperone and plays a decisive role in the maintenance of eye lens transparency, however, in vitro carbamylation of the α-crystallin through a high-molecular-weight aggregates formation causes loss of its chaperone activity [176].

### Degenerative protein modifications and its validation

In DPMs validation, the commercial specific antibodies for quantifying DMPs are very limited. Proteomics technique like multiple reactions monitoring (MRM) can be used for validation of DPMs, however, instruments with low resolution (i.e. <2000 resolution) cannot differentiate deamidated and unmodified peptides [52]. High resolution (70,000–140,000) instrument like Q-Exactive can theoretically differentiate deamidated and unmodified ions [177]. When triple quadrupole mass spectrometers collects precursor and product ions for validation of deamidated peptides, the ^13^C peaks of their undeamidated counterparts are also collected by the commonly used isolation width of 0.7 Th [48]. Thus, it’s not possible to validate DPMs like deamidation. Since DPMs occurs spontaneously either in vivo or in vitro, and produces a mixture of both modified and unmodified products, the potential to differentiate between them using high-resolution PRM remains critical for the accurate quantification of protein modifications in biological systems. Thus, although proteomics technology is well developed for identification of DPMs but still recent advances cannot help to validate it and needs much more efforts to develop required methods. The biological functions of spontaneous DPMs is not easy to validate in vitro or in vivo. For example, oxidative damaged proteins are proposed and known to induce natural aging and degenerative diseases for decades, but direct proof of their biological functions are not possible. Intriguingly, protein N-deamidation can be promoted by microenvironmental factors such pH and oxidative stress [178], as well as protein interactions with other molecules and spontaneous mutations that alter protein sequence [179–181]. Although N-deamidation is a spontaneous process, host cells employ a repair mechanism that prevents the excessive accumulation of deamidated residues in proteins. This repair mechanism is mediated by the enzyme L-isoaspartyl (D-aspartyl) methyltransferase (PIMT) [19], which is most highly expressed in mammalian brain tissues [182], and exhibits decreasing activity in aging mice [183]. Accordingly, PIMT-deficient mice suffer neuropathology and fatal epileptic seizures at 30-60 days after birth [184, 185]. These experiments using transgenic animal models clearly demonstrated that spontaneous degenerative protein N-deamidation and the action of its repairing PIMT critically influence the structure and function of key proteins in the central nervous system.

### Evaluating protein expression and specific DPMs as potential biomarkers of dementia

The early diagnosis of dementia and development of effective new drug therapies will depend on the identification of robust biomarkers of disease pathology. However, previous efforts to uncover clinically useful prognostic/diagnostic biomarkers in dementia have been restricted by an incomplete understanding of the mechanisms underpinning disease pathogenesis. Earlier attempts to identify disease biomarkers have primarily been guided by the ‘amyloid hypothesis’ or Tau-based models of dementia pathogenesis, hence these have largely focused on the components of extracellular amyloid plaques, intraneuronal NFT, and on Tau isoforms in the brain. Given that sampling of human brain tissues is poorly suited to routine clinical testing, many biomarker studies have instead focused their analyses on CSF, which benefits from close proximity to the brain extracellular space and is known to reflect biochemical and molecular changes that occur within the brain parenchyma. Accordingly, the most clinically useful biomarkers identified to date are CSF levels of Aβ_1–42_, total Tau protein, and phospho-Tau-181, which are significantly elevated in AD [186, 187], but are unable to differentiate other forms of dementia. Other proposed biomarkers of AD have included CSF accumulation of proteins such as phospholipase A2, visinin-like 1, and various neurofilament components (recently reviewed by Liu et al. [188]). Another possible biomarker of AD pathogenesis is increased generation of the CSF component acid 2,4-dihydroxybutyrate, which has previously been shown to correlate with the progression of MCI [83, 189]. However, a more promising approach might be the development of complex panels of multiple biomarkers that can provide a more detailed picture of pathological events occurring in the brain. Indeed, in a recent study of human CSF samples, Shi et al. used a targeted approach to develop a biomarker profile capable of distinguishing patients with PD and AD by detecting dysregulated levels of macrophage colony-stimulating factor 1 receptor, osteopontin (SPP1), pro-low-density lipoprotein receptor-related protein 1, ephrin type-A receptor 4, and metalloproteinase inhibitor 1 [190].

Brain synapses play major roles in neuronal communication and their dysfunction is associated with cognitive disturbance in early AD. Given that synaptic dysfunction is thought to occur prior to neuronal cell degeneration and death, it is possible that proteins expressed in synapses could serve as very early biomarkers of disease pathogenesis. A promising candidate is neurogranin protein which participates in synaptic signaling events via the regulation of calmodulin availability and is also known to be involved in long-term potentiation and memory consolidation [191]. Neurogranin is abundantly expressed in the cerebral cortex, hippocampus, amygdala, and striatum, and elevated levels of this protein in CSF predict AD progression and rapid cognitive deterioration [192]. The presynaptic protein SNAP25 has also been detected at significantly increased levels in CSF from AD patients [193]. However, CSF collection by lumbar puncture remains an invasive method with high risk and significant side effects, hence there is a clear unmet need for more readily accessible biomarkers in other body fluids that are better suited to longitudinal analyses of individual patients over an extended period.

The biological fluids most easily sampled in clinical settings are blood and urine, hence mass spectrometry-based proteomics has been widely used in previous attempts to identify blood biomarkers of dementia [194]. An altered ratio of Aβ42:Aβ40 in plasma [195], and increased serum levels of proteins including ApoE [196], clusterin [197], α-1-antichymotrypsin [198], and cytokines IL-1α and IL-6 [199] have been proposed, but the clinical utility of these for differentiating dementia subtypes and disease stages has not been established, and concerns over the sensitivity and specificity of these putative biomarkers remain unresolved. Indeed, an increase in circulating levels of Aβ has been reported in familial AD and Down syndrome, this finding was not replicated in sporadic AD, and plasma levels of Aβ_1–42_ and Aβ_1–40_ have been variably reported as either elevated or reduced, or even unchanged in AD patients compared with controls [200, 201]. These findings suggest that blood concentration of Aβ is unlikely to represent a robust biomarker for clinical applications. Similarly, while Ray et al. [202] were able to identify a panel of 18 signaling proteins that achieved almost 90 % accuracy in identifying MCI patients that later progressed to AD, this strategy later failed cross-validation on an independent assay platform. Several other researchers have also attempted to identify AD patients by assessing plasma levels of serotonin, phenylalanine, proline, lysine, phosphatidylcholine, taurine and acylcarnitine; metabolites including phospatidylinositol, proline-asparagine dipeptide, acylcarnitines, malic acid, lysophophatidylcholine, and glycoursodeoxycholic acid: and various combinations of circulating proteins, lipids, metabolites and other blood biomolecules [203, 204]. While encouraging early data have been generated by combinations of lipidomic, proteomic and metabolomic approaches, the results from these studies have so far not been replicated in independent clinical cohorts and will require further investigation [205].

Increased protein damage by DPMs like glycation, oxidation and nitration has been implicated in neuronal cell death leading to AD. Ahmed et al. [206] measured glycation, oxidation and a nitration adduct in CSF samples of AD and age-matched control and found increased concentration of 3-nitrotyrosine, Ne-carboxymethyl-lysine, 3-deoxyglucosone-derived hydroimidazolone and N-formylkynurenine residues. Although protein nitration, oxidation and glycation adducts in CSF have been proposed as a biomarker, the variables linked to these modifications may also be useful indictors for the diagnosis of AD. Pentosidine, an advanced glycation end product could be an important factor useful for the diagnosis of AD [207]. Conrad et al. [208] found significant elevation of total oxidized plasma proteins in AD subjects when compared with non-AD controls and suggested that such oxidized proteins may be useful as biomarkers for the detection and evaluation of AD. However, their study focused on total oxidized proteins and not attempted to identify individual proteins. Oxidized β-amyloid in CSF has been proposed to be a biomarker to differential subjects with Lewy body dementia from patients with PD dementia [209], while oxidized plasma fibrinogen γ-chain precursor proteins and α-1-antitrypsin [210] were relevant to diagnosis of AD. Several studies have found increased oxidizablility of CSF\plasma derived lipoproteins and APOA-I [211, 212].

### Exosomes as novel biomarkers of neurodegenerative diseases

Although blood is a rich source of potential disease biomarkers, detecting rare circulating proteins against a high background of extremely abundant proteins such as albumin can be very challenging. Accordingly, efforts are underway to develop methodologies that can remove highly abundant proteins from blood without depleting disease-relevant molecules with potential clinical utility. However, an alternative strategy for identifying blood biomarkers of dementia could be to move away from analysis of soluble proteins to assessing the contents of circulating extracellular vesicles (EVs). EVs include a wide range of structures derived from the plasma membrane or endosomal origins. These vesicles are released by almost all cell types and play major roles in many critical biological processes (including intercellular communication, myelination, synaptic plasticity, antigen presentation, trophic support of neurons, tissue repair, immune surveillance, and blood coagulation) [213–217]. EVs are released under both normal and pathological conditions, and have been detected in body fluids as diverse as saliva, breast milk, amniotic fluid, hydrothoracic fluid, and ascitic fluid [218]. While these vesicles are only 30–50 nm in diameter, they contain a complex cargo of proteins, lipids, and various RNA species derived from the host cell of origin [219]. Consequently, EVs are laden with biomolecules that reflect pathophysiological conditions in the tissue from which they were originally released, and are therefore regarded as an extremely promising source of circulating biomarkers for use in clinical diagnostics. Due to their small size and low density, EVs (including exosomes) have typically been isolated for study via differential ultracentrifugation. However, Gallart-Palau et al. [220, 221] recently reported a novel method of enriching EVs from human plasma and brain tissues via Protein Organic Solvent PRecipitation (PROSPR), which could significantly accelerate progress in this field.

The clinical potential of assessing EV cargo is well supported by previous reports that exosomes derived from the brain and CSF of AD patients are enriched in Aβ peptides and phosphorylated Tau [222, 223]. In addition, exosomal transfer of α-synuclein protein has been proposed to contribute to the pathogenesis of PD, and these vesicles may also be involved in the dissemination of prion proteins by ‘infected’ neuronal cells [224, 225]. The trafficking of macromolecules from the central nervous system (CNS) to the CSF and blood can also be mediated by EVs [219], and CNS-derived vesicles have been successfully detected in both CSF and blood serum [226]. The studies conducted to date have already identified that exosomes are laden with numerous proteins that are associated with neurodegenerative pathology, including APOE, Aβ peptides, α-synuclein, prion, and neurogenic locus notch homolog protein 3 (NOTCH3) [88, 225, 227–229]. Accordingly, exosomes enriched in Aβ and α-synuclein has been reported to impair neuronal cell survival and potentially contribute to the pathogenesis of AD [227]. It is important to note that the clinical potential of exploiting EV biology is not restricted to dementia alone. For example, serum exosomes from glioblastoma patients have been shown to contain glioblastoma-specific epidermal growth factor receptor vIII (EGFRvIII) which promotes tumor growth and may represent a useful diagnostic biomarker of glioblastoma [230]. Other investigators have also reported that tumor cells secrete vesicles that can modulate the microenvironment to facilitate angiogenesis and metastasis [231]. Indeed, the clinical exploitation of exosome biology may also yield significant advances in prognostic testing of patients with melanoma, ovarian cancer, bladder cancer, prostate cancer, kidney injury or liver damage [230, 232–234].

### Future outlook

Dementia is now a global public health priority and will require urgent action to address at both healthcare and societal levels. Defining the molecular mechanisms underlying dementia pathology will be key to developing an effective cure, but this is unlikely to be achieved using classical biological methods that focus only on the role of a select few genes in disease pathogenesis. Researchers now appreciate that neurodegenerative disorders arise from complex interactions between a wide ranges of proteins, and that advanced proteomics technologies will be required to identify and quantify disease-related protein profiles with prognostic value for use in the clinic. As outlined in this review, unbiased, global, discovery-driven approaches such as proteomics are well-suited to uncovering the complex molecular pathology of human proteinopathies such as dementia. State-of-the art quantitative proteomics techniques are now capable of accurately profiling the human brain proteome and dissecting complex vesicular cargoes, leading to a new appreciation that DPMs play critical roles in altering protein function, aggregation and deposition in disease. Translating this recently acquired knowledge into new clinical applications will be a major challenge in the years ahead. Indeed, future studies will need to employ targeted proteomics alongside discovery-based approaches in order to fully elucidate dementia pathology and enable the development of novel therapies for affected patients. Obtaining well-characterized tissues from specific brain regions remains a major barrier to progressing the field, but future advances will also require the implementation of targeted government policies and proteomics funding schemes that can help researchers translate recent technological advances into novel clinical applications.

## Abbreviations

AD: Alzheimer’s disease
AD-CVD: Alzheimer’s disease with cerebrovascular diseases
AGEs: Advanced glycation end products
ALS: Amyotrophic lateral sclerosis
Apo E: Apolipoprotein E
Aβ: β-amyloid
CBD: Corticobasal degeneration
CK: Creatine kinase
CNS: Central nervous system
CSF: Cerebrospinal fluid
DLB: Dementia with lewy bodies
DPMs: Degenerative protein modifications
EALIC: Electrostatic attraction hydrophilic interaction chromatographic mode
emHILIC: Electrostatic-interaction modified HILIC hydrophilic interaction liquid chromatography
EVs: Extracellular vesicles
FTD: Frontotemporal dementia
GABA: γ-aminobutyric acid
GAPDH: Glyceraldehydes 3-phosphate dehydrogenase
GS: Glutamine synthetase
HIF: Hypoxia-inducible transcription factor
HILIC: Hydrophilic interaction liquid chromatography
IPL: Inferior parietal lobule
iTRAQ: Isobaric tags for relative and absolute quantitation
LDH: Lactate dehydrogenase
MBP: Myelin basic protein
MCI: Mild cognitive impairment
miRNA: micro RNA
mRNA: Messenger RNA
NFTs: Neurofibrillary tangles
NT: 3-nitrotyrosine
PD: Parkinson’s disease
PGM1: Phosphoglycerate mutase 1
PHFs: Paired helical filaments
PIMT: Protein ‘L-isoaspartate (D-aspartate) O-methyltransferase’
PROSPR: Protein Organic Solvent Precipitation
PSEN1: Presenilin 1
PSEN2: Presenilin 2
RAGE: Receptor advanced glycation end products
RNS: Reactive nitrogen species
ROS: Reactive oxygen species
SAX: Strong anion exchange
SDC: Sodium deoxycholate
SNAP25: Synaptosomal-associated protein 25
TMT: Tandem mass tag
TPI: Triose phosphate isomerise
UC-ERLIC: Ultracentrifugation-electrostatic repulsion hydrophilic interaction chromatography
UCH L-1: Ubiquitin carboxy-terminal hydrolase L-1
VaD: Vascular dementia
VDAC: Voltage dependent anion channel
WAX: Weak anion exchange
WHO: World Health Organization
